# Do public employment services affect the self-rated health of migrant workers in China?

**DOI:** 10.1371/journal.pone.0270006

**Published:** 2022-07-08

**Authors:** Lilian Li, Bingxue Xu, Chunyan Chen, Mingwang Cheng

**Affiliations:** 1 School of Economics, Jiangxi University of Finance and Economics, Nanchang, 330013, China; 2 School of Economics and Management, Tongji University, Shanghai, 200092, China; 3 Department of Gynaecology and Obstetrics, Anting Hospital, Jiading District, Shanghai, 201805, China; 4 Institute of Economic Research, Dali University, Dali, 671003, China; University of Jyvaskyla, FINLAND

## Abstract

Migrant workers greatly contributing to China’s industrialization and urbanization are confronted with increasing health risks. This study empirically investigates the effects of public employment services on the self-rated health of migrant workers in Shanghai China, by using data from the National Bureau of Statistics from 2015 to 2020. The estimation results under the Ordered Probit model illustrate that public employment services significantly improve the self-rated health of migrant workers, and vocational training, job development and other related services show an apparently positive correlation with the self-rated health. The marginal effect analysis reveals that public employment services obviously reduce the probability of health satisfaction as “average”, “relatively satisfied” and “relatively dissatisfied”, which translate into a significant increase in the probability of “very satisfied”. The mechanism analysis verifies that public employment services enhance the self-rated health by increasing the proportion of medical insurance and injury insurance of migrant workers. The results are still reliable by adopting the methods of subsample regression, Propensity Score Matching and variable substitution to conduct robustness checks. This study further enriches the literature on public employment services and the health status of migrant workers, and provides policy implications on improving the health status of migrant workers and the public employment service system of China under the impact of the COVID-19 pandemic.

## 1. Introduction

Since the reform and opening-up, China has formed the largest population of migrant workers in the world with rapid urbanization and industrialization [1]. At the end of 2019, the number of migrant workers in China reached 290.77 million, accounting for 37.5% of the working-age population, which contributes greatly to China’s economic growth [2]. However, due to the restriction of China’s unique *hokou* (household registration) system, migrant workers do not obtain the same benefits and services as local urban residents, including employment, medical services and minimum living allowance [3–8]. Moreover, migrant workers are confronted with the increased risks of physical and mental health caused by high-intensity work, environmental pollution, lack of social security and long-term separation with family [9,10]. In addition, affected by the outbreak of COVID-19, the Chinese government took the epidemic prevention measures of locking down high-risk areas, which had a negative impact on the employment of migrant workers. The number of migrant workers in 2020 had a prominent decline by 5.17 million compared with 2019. As a result, migrant workers are facing with the decreasing medical expenditure because of lack of employment opportunities and source of income, which is detrimental to the health conditions of migrant workers. Therefore, to explore the health status of migrant workers is of great practical significance for improving their happiness and for promoting China’s urbanisation and high-quality development.

Indeed, scholars have conducted extensive studies on the health issues of migrant workers producing abundant research results. First, some studies have confirmed the “healthy migration effect” [11–14], which states that healthy labourers are more willing to migrate. It means that the health status of migrant workers is generally higher than that of local residents, but the health advantages of migrant workers would gradually disappear with the work and life in destination. Second, education is deemed as the crucial human capital factor to improve the health conditions of migrant workers [15]. Migrant workers with higher education can be more likely to obtain high-income, healthy and safe jobs which enhances the medical affordability and health security for migrant workers, and education can also raise the awareness of their health management [16,17]. Third, several studies have explored the effect of social capital on the health status of migrant workers, which illustrates that social capital improves the health status of workers through mutual assistance, emotional support and information sharing of social network members, such as food, housing, medical assistance, emotional communication and health knowledge sharing [18,19]. Furthermore, some scholars have investigated the health issues of migrant workers from the perspectives of work environment, acculturation, urbanisation, discrimination and the Internet [20–26].

However, existing studies have not addressed the effects of public employment services on the health status of migrant workers in China. The public employment service system is designed to improve the matching efficiency of labour market; hence, existing studies have paid extra attention to the employment effects of the public employment services of migrant workers [27–29]. Mortensen and Pissarides [30] expounded the process of job creation and destruction in the labour market and deemed that market heterogeneity, factor flow cost and incomplete information result in unemployment. Then, the DMP model constructed by Diamond [31], Pissarides [32] and Mortensen [33] systematically elaborates the theoretical relationship among the formation of unemployment, job search and job vacancy that forms the classical search-matching theory, for which they jointly won the Nobel Prize in Economics in 2010. The important applications of search-matching theory in the field of public policy are that the government should establish the public employment service system to strengthen labour market management, reduce the search costs of job seekers and improve the matching efficiency between job seekers and vacancies. Most studies have argued that public employment services, such as counselling, vocational guidance and skills training, increase the employment of job seekers [34–36]. Migrant workers participating in public employment services may obtain more employment opportunities, which have potentially positive effects on their health conditions. First, the participants may get high-paying jobs, which conduce to improving their affordability for medical service. Second, the participants may obtain jobs with healthier environment and better medical security relative to the non-participants. Third, the participants may raise their health awareness and concern whether employers provide various medical insurances. The reliable evidence shows that medical service and healthier work environment are positively relevant to the health status of migrant workers [37,38]. It implies that the mechanism of public employment services on the health status of migrant workers exists.

Hitherto, the existing study has rarely discussed the relationship between public employment services and the health status of migrant workers in China. This research is the first to provide empirical evidence to examine the impacts of public employment services on the self-rated health of migrant workers in China. There are three main reasons for taking China as the research object. First, China has the largest number of migrant workers in the world with 290.77 million migrant workers at the end of 2019. The health conditions of migrant workers are related to China’s urbanisation process, economic growth and social stability. Moreover, Shanghai is a typical city that inputs migrant workers and had 9.78 million migrant population in 2020, accounting for 40.27% of the city’s total population, for which the policy implications have reference values for other places in China to deal with the problems of migrant workers. Third, although China’s Employment Promotion Law enacted in 2008 has clearly defined the functions of public employment service agencies, whether the public employment service system can safeguard migrant workers’ health is unclear. Hence, this study utilises the data from the dynamic monitoring survey of migrant workers conducted by the Shanghai survey team of the National Bureau of Statistics from 2015 to 2020. It also investigates the effects of public employment services on the self-rated health of migrant workers.

The contributions of this paper are threefold relative to existing studies. First, it is the first to confirm the positive effects of public employment services on the self-rated health of migrant workers on the basis of search-matching theory. Second, this work provides reliable empirical evidence from the perspectives of the coverage, depth and heterogeneity of public employment services, which adopt the techniques of Ordered Probit Model, Propensity Score Matching (PSM) and a series of robustness tests. Third, mechanism analysis indicates that public employment services improve the self-rated health by increasing the proportion of medical insurance and injury insurance of migrant workers. Last, this study further enriches the literature on public employment services and the health status of migrant workers. Policy implications on improving the health status of migrant workers and the public employment service system of China are also provided.

## 2. Data and methods

### 2.1 Data source

The data in this study was obtained from the dynamic monitoring survey on the process of migrant workers’ citizenization conducted by the Shanghai investigation team of the National Bureau of Statistics from 2015 to 2020. The survey systematically investigated the basic situation of migrant workers in Shanghai, covering individual or family characteristics, employment status, health and medical treatments, social integration, children’s education, family income and expenditure, living conditions and residence intention. During the survey, the goals of the study were explained to respondents, and they were informed that participation was voluntary and anonymous. Filling in the questionnaire was considered to constitute informed consent. The data was anonymized before access, the personal information (e.g. name, id) was stripped, and none of the authors of this manuscript were involved in the collection of these data. After excluding the samples with missing data in 2019, we have collected 6,064 samples in total. The samples ranged in age from 16 to 77, including 3,601 male samples and 2,463 female samples. There were 5,565 employment samples and 499 unemployment samples. Given that the study mainly focuses on the samples of employees, we finally obtained 4,788 valid samples by excluding those of unemployment, employers and the self-employment of migrant workers. Since the random sampling is employed to collect data in the dynamic monitoring survey every year, our study uses the mixed cross section data. According to the national regulations of China, the ethical approval of the Chinese Ethics Committee is compulsory for biomedical research. Because this study was not biomedical but an organizational survey research with no minors involved, ethical approval was not necessary.

### 2.2 Variable setting

#### 2.2.1 Dependent variable

The measurement of the health status of migrant workers that includes physical and mental health. Physical health can be measured by self-rated health, physical examination indicators and Hopkins Symptomatic Checklist [39,40]. Meanwhile, mental health can be measured by Symptom Check List-90 (SCL-90), Center for Epidemiologic Studies Depression Scale (CES-D), and subjective health evaluation [41–43]. The dependent variable, self-rated health, is measured by respondents’ subjective evaluation on their health satisfaction (denoted by HS) [37,42], which ranges from “very dissatisfied” = 1, “relatively dissatisfied” = 2, “average” = 3, “relatively satisfied” = 4 and “very satisfied” = 5. The higher the score of the dependent variable, the higher the health satisfaction of migrant workers.

#### 2.2.2 Explanatory variable

The core explanatory variables are defined by seven indicators from three perspectives of the coverage, depth and heterogeneity of public employment services. In terms of coverage, PES is set as a dummy variable defined as “Have you received public employment services? Yes = 1, No = 0”. In terms of depth, TPES is referred to as “How many types of public employment services have you received?” and ranges from 0 to 5. In terms of heterogeneity, VI is defined as “Have you received vocational introduction? Yes = 1, No = 0”. VG is referred to as “Have you received vocational guidance? Yes = 1, No = 0”. Vocational training (VT) is defined as “Have you received vocational training? Yes = 1, No = 0”. Job development (JD) is referred to as “Have you received job development? Yes = 1, No = 0”. The variable of other related services (ORS) is defined as “Have you received any other employment services? Yes = 1, No = 0”. Moreover, the control variables are the following factors: 1) individual characteristics, such as gender, age, marriage, household registration, education, residence permit and political identity; 2) economic and social characteristics, including trailing relatives and medical insurances; 3) characteristics of domicile place, including east, middle and west, where the northeast is the control group; 4) industry characteristics, including manufacture and service in which agriculture is the control group. The definitions of variables for further details display in Table 11 in the Appendix.

### 2.3 Estimation method

#### 2.3.1 Benchmark model

The dependent variable, self-rated health (HS) of migrant workers, is a discrete ordered variable from 1 to 5; hence, the benchmark regression in this research is estimated by applying the Ordered Probit Model. The specific model form is as follows:

$$\mathrm{Probit}(HS_{it}^{*})=\alpha +\beta X_{it}+\gamma CV_{it}+\varepsilon_{it}$$ (1)

where HS* is the latent variable of HS, representing the self-rated health of individual *i* in year *t*. X represents a set of core explanatory variables, including PES, TPES, VI, VG, VT, JD and ORS. In addition, β is the estimated parameter of the core explanatory variables that we are most interested in. When β is significantly greater than 0, a positive correlation exists between public employment services and self-rated health. CV is a series of control variables with potential impacts on the self-rated health of migrant workers, and *ε* is the error term. In addition, HS* and HS follow the relationship:

$$HS_{it}=\{\begin{array}{l} =1,\;HS_{it}^{*}\leq R_{1}\\ =2,\;R_{1}<HS_{it}^{*}\leq R_{2}\\ =3,\;R_{2}<HS_{it}^{*}\leq R_{3}\\ =4,\;R_{3}<HS_{it}^{*}\leq R_{4}\\ =5,\;R_{4}<HS_{it}^{*} \end{array}$$ (2)

where *R*_*1*_*–R*_*4*_ are the parameters (cut-off points) to be estimated and satisfy *R*_*1*_ *< R*_*2*_ *< R*_*3*_ *< R*_*4*_. Then, we further estimate the marginal effects of public employment services on self-rated health, which denotes the probability of the impacts of public employment services on HS.

#### 2.3.2 Methods for the robustness check

To testify the reliability of the estimate results of the benchmark model, this study adopts the methods of subsample regression, variable substitution and Propensity Score Matching (PSM) to conduct robustness checks [44–47].

First, this study divides the sample into two groups by age, sex, and marriage for performing the subsample regression. Because the old (born before 1980), female and married migrant workers are the vulnerable groups in the labour market, their health satisfaction may be lower due to their disadvantages in labour endowment, gender discrimination or intra-household labour division. The study attempts to discuss whether there are different effects of public employment services on self-assessment of health among different groups, which can also be used to verify the robustness of the benchmark regression results.

Second, we further employ the recognizing strategy of variable substitution for robustness test. Specifically, the explained variable, health satisfaction of migrant workers, is redefined as “Are you very satisfied with your health? Yes = 1, No = 0” (denoted by HS1), which is a dichotomous dummy variable. Then, we apply the Probit Model for the regression analysis and calculate the marginal effect of the estimated coefficient.

Third, considering that migrant workers receiving the public employment services may be the result of “self-selection”, e.g., migrant workers with low education levels may be more interested in receiving in public employment services provided free by the government because of their limitations in employment information and competitiveness. Therefore, we use the PSM method to test the robustness for the benchmark model to reduce the selection bias, which can also avoid the potential estimation bias caused by improper model setting. The core thought of PSM is to identify the causal effect by constructing the counterfactual framework of public employment service on self-rated health of migrant workers. In general, the causal effect of PSM is called the average treatment effect on treated (ATT), and the mathematical expression of ATT takes as follow:

$$\begin{array}{l} \mathrm{ATT}=E(HS_{1i}-HS_{0i}|D_{i}=1)=\underset{\mathrm{treatment}\;\mathrm{group}}{\underset{︸}{E(HS_{1i}|D_{i}=1)}}-\underset{\mathrm{counterfactual}\;\mathrm{results}}{\underset{︸}{E(HS_{0i}|D_{i}=1)}}\\ \;=\underset{\mathrm{Difference}\;\mathrm{between}\;\mathrm{treatment}\;\mathrm{and}\;\mathrm{control}\;\mathrm{group}}{\underset{︸}{E(HS_{1i}|D_{i}=1)-E(HS_{0i}|D_{i}=0)}}+\underset{\mathrm{Selection}\;\mathrm{bias}}{\underset{︸}{E(HS_{0i}|D_{i}=0)-E(HS_{0i}|D_{i}=1)}} \end{array}$$ (3)

where D_i_ is a dummy variable, the D = 1 and D = 0 respectively represent treatment group and control group. The AAT is equal to the difference between the self-rated health for the treatment group and its counterfactual results, which can be further decomposed into two parts of the selection bias and the difference of HS between treatment group and control group. Finally, we ensure the selection bias is equal to zero by using the balance test, then the ATT is equal to the difference between treatment group and control group.

#### 2.3.3 Mediating effect model

In fact, migrant workers receiving public employment services are more likely to get jobs with medical insurance (MI) and injury insurance (II), which will have a positive effect on migrant workers’ self-rated health. Therefore, the MI and II may be the potential mechanism through which public employment services affect the self-rated health of migrant workers, this study uses an intermediate effect model to test whether this mechanism exists [48]. The model is set as follows:

$$Channel_{it}=\alpha_{0}+k_{1}X_{it}+\gamma_{1}CV_{it}+\varepsilon_{it}$$ (4)

$$HS_{it}^{*}=k_{2}X_{it}+\lambda Channel_{it}+\gamma_{2}CV_{it}+\varepsilon_{it}$$ (5)

$$HS_{it}^{*}=(k_{2}+\lambda k_{1})X_{it}+(\gamma_{2}+\lambda \gamma_{1})CV_{it}+\varepsilon_{it}$$ (6)

where *Channel* is the mediator, MI and II respectively. A variable may be considered a mediator when (1) the explanatory variable X significantly affects the mediator, and (2) the X significantly influences the dependent variable *HS* taking no account of the mediator, and (3) the effect of the mediator on the *HS* is significantly upon the addition of the X to the model [49]. Therefore, the conditions for the existence of mediating effects are that the k_1_, k_2_ and k_2_+λk_1_ are significantly non-zero.

## 3. Results

### 3.1 Statistical analyses

Table 1 shows the statistical descriptions of the main variables. Specifically, 25% of migrant workers participate in public employment services, and migrant workers participate in 0.61 public employment service on average (TPES = 0.61). The proportion of migrant workers participating in VI, VG, VT, JD and ORS accounts for only 16%, 16%, 19%, 4% and 6%, respectively. In addition, the average self-rated health of the total sample is 4.10, and the HS of participants (receiving PES) is 4.17, which is higher than that of non-participants (HS = 4.06). The young, unmarried and higher-educated migrant workers are more willing to receive PES than those of older, married and lower-educated migrant workers. Migrant workers engaged in manufacturing industry have stronger motivation to participate in PES, while migrant workers engaged in service industry have weaker motivation to participate in PES.

**Table 1:** Descriptive statistics of the main variables.

Variable	Total sample		Participants(PES = 1)		Non-participants(PES = 0)	
	Mean	SD	Mean	SD	Mean	SD
HS	4.10	0.66	4.18	0.66	4.07	0.66
PES	0.25	0.43				
TPES	0.61	1.25	2.46	1.31		
VI	0.16	0.36	0.63	0.48		
VG	0.16	0.36	0.63	0.48		
VT	0.19	0.40	0.78	0.42		
JD	0.04	0.21	0.18	0.38		
ORS	0.06	0.24	0.25	0.43		
Gender	0.59	0.49	0.62	0.48	0.58	0.49
Age	37.51	10.74	35.84	10.08	38.06	10.90
Marriage	0.82	0.38	0.78	0.42	0.83	0.37
Household registration	0.96	0.19	0.96	0.20	0.96	0.19
Education	10.57	3.31	11.11	3.20	10.39	3.32
Residence permit	0.62	0.48	0.62	0.49	0.62	0.48
Political identity	0.02	0.16	0.03	0.16	0.02	0.15
Trailing relatives	0.03	0.18	0.02	0.14	0.04	0.19
Medical insurance	0.68	0.45	0.76	0.41	0.65	0.47
East	0.34	0.47	0.34	0.47	0.34	0.47
Middle	0.50	0.50	0.52	0.50	0.49	0.50
West	0.15	0.35	0.12	0.33	0.15	0.36
Manufacture	0.32	0.46	0.41	0.49	0.28	0.45
Service	0.60	0.49	0.54	0.50	0.61	0.49

**Fig 1:**
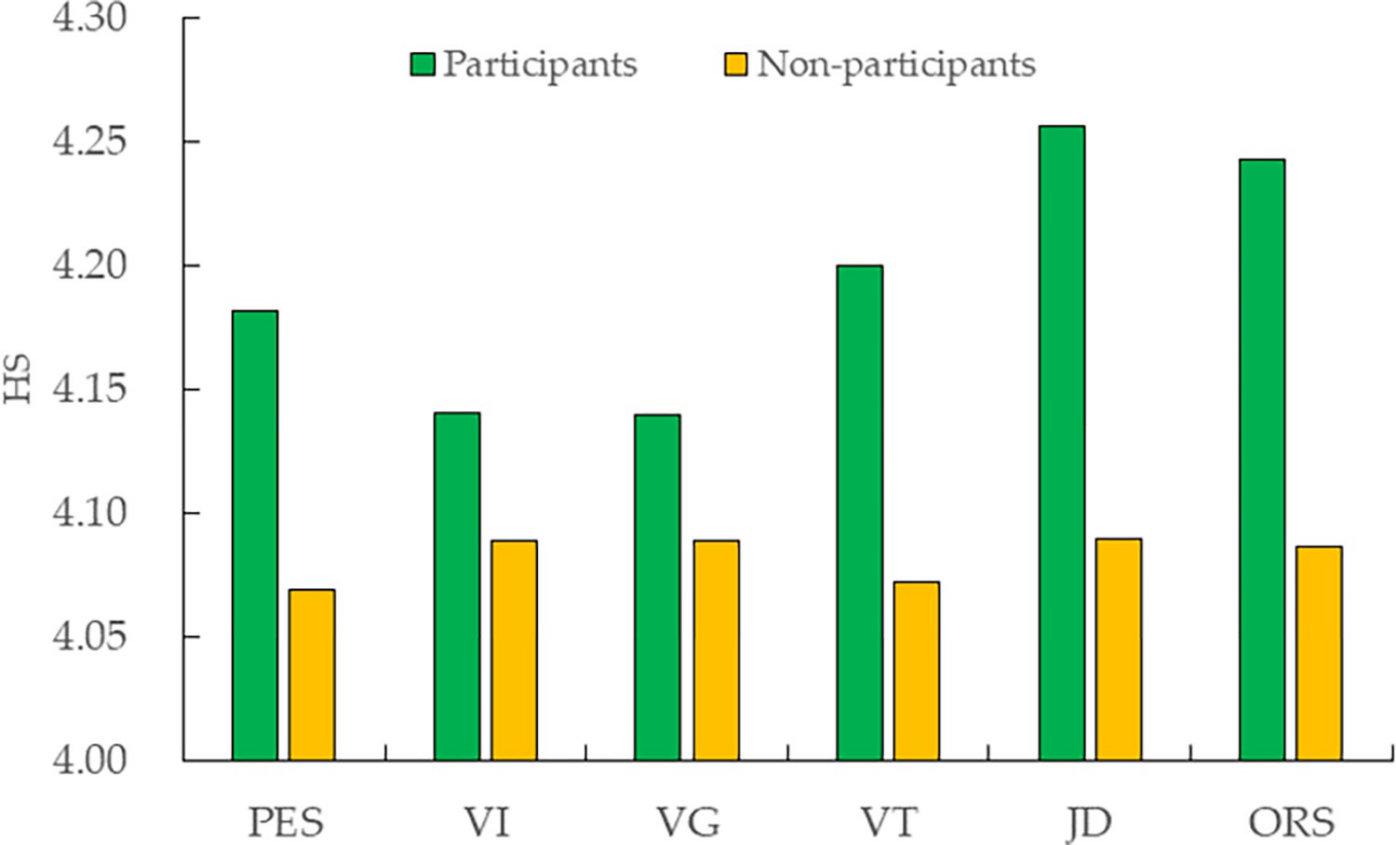
Differences in HS between participants and non-participants. Data is from the National Bureau of Statistics.

Fig 1 displays the differences in self-rated health between participants and non-participants. The average HS of participants receiving PES is 4.182, which is apparently higher than that of 4.069 for non-participants; the difference in HS between participants and non-participants is 0.113. In terms of the types of public employment services, the average HS of participants who participate in VI, VG, VT, JD or ORS is apparently higher than that of non-participants. Overall, the HS of participants is obviously higher than that of non-participants, indicating that public employment services may improve the HS of migrant workers. Specifically, a prominent improvement is observed in the HS of migrant workers who participate in VT, JD or ORS. The reliability of this conclusion needs to be further verified by conducting regression analysis, controlling other interfering factors in the following.

### 3.2 Benchmark regression results

To verify the reliability of the above consequences, we apply the Ordered Probit Model for empirical test. The estimated results of the benchmark regression are shown in Table 2. Column (1) examines the effect of PES on the HS of migrant workers, adding the main control variables. The estimated coefficient of PES is significantly positive (*p* < 0.01), indicating that the HS of migrant workers participate in PES is higher than that of migrant workers not participating in PES. Column (2) investigates the effect of TPES on the HS, and the estimated coefficient is positive (*p* < 0.05), which illustrates that the HS of migrant workers is higher with more types of public employment services participated. Columns (3)–(7) further explore the impacts of different types of public employment services on the HS of migrant workers. Among them, the estimated coefficients of VT, JD and ORS are significantly positive (*p* < 0.01), suggesting that these three types of public employment services significantly improve the HS of migrant workers. However, VI and VG have positive but insignificant effects on the HS, which is no clear evidence that VI and VG distinctly improve the HS of migrant workers. Therefore, the positive effects of public employment services on the HS of migrant workers are mainly from VT, JD and ORS.

**Table 2:** Public employment services and self-rated health.

Variable	Dependent variable: HS						
	(1)	(2)	(3)	(4)	(5)	(6)	(7)
PES	0.116***						
	(0.037)						
TPES		0.031**					
		(0.013)					
VI			0.011				
			(0.043)				
VG				0.019			
				(0.043)			
VT					0.148***		
					(0.040)		
JD						0.258***	
						(0.078)	
ORS							0.179***
							(0.067)
Control variable	YES	YES	YES	YES	YES	YES	YES
*N*	4788	4788	4788	4788	4788	4788	4788

Robust standard errors are in parentheses

*, ** and *** denote significance at the 10%, 5% and 1% level, respectively. Data is from the National Bureau of Statistics.

The implication of the estimated coefficient of the Ordered Probit Model is not intuitive. We only obtain the coefficient symbols and significance. Hence, we further calculate the marginal effects of public employment services on different levels of the HS of migrant workers, that is, the probability change of each value of explained variable caused by 1 unit change of explanatory variable. The results of the marginal effects are shown in Table 3. Column (1) displays the marginal effects of PES on different levels of HS. The marginal effect of PES on HS as “very satisfied” is 0.038 (*p* < 0.01), manifesting that the probability of “very satisfied” of migrant workers receiving PES increases by 3.8%. In addition, the marginal effects of PES on the HS as “relatively satisfied”, “average” and “relatively dissatisfied” are −0.011, −0.025 and −0.002 (*p* < 0.01), which illustrate that the probability of “relatively satisfied”, “average” and “relatively dissatisfied” of migrant workers receiving PES decrease by 1.1%, 2.5% and 0.2%, respectively. Similarly, Column (2) implies that the probability of “very satisfied” of migrant workers receiving TPES increases by 1.0% and the probability of “relatively satisfied”, “average” and “relatively dissatisfied” decrease by 0.3%, 0.6% and 0.1%, respectively.

**Table 3:** Marginal effects of public employment services on self-rated health.

Different levels of HS	Explanatory variables						
	PES	TPES	VI	VG	VT	JD	ORS
	(1)	(2)	(3)	(4)	(5)	(6)	(7)
very dissatisfied	-0.001 (0.001)	-0.000 (0.000)	-0.000 (0.000)	-0.000 (0.000)	-0.000 (0.000)	-0.000 (0.000)	-0.000 (0.000)
relatively dissatisfied	-0.002*** (0.001)	-0.001** (0.000)	-0.000 (0.001)	-0.000 (0.001)	-0.003*** (0.001)	-0.004*** (0.001)	-0.003*** (0.001)
average	-0.025*** (0.008)	-0.006** (0.003)	-0.002 (0.009)	-0.004 (0.009)	-0.031*** (0.008)	-0.055*** (0.016)	-0.038*** (0.014)
relatively satisfied	-0.011*** (0.004)	-0.003*** (0.001)	-0.001 (0.004)	-0.002 (0.004)	-0.014*** (0.004)	-0.025*** (0.008)	-0.017*** (0.005)
very satisfied	0.038*** (0.012)	0.010*** (0.004)	0.003 (0.014)	0.006 (0.014)	0.048*** (0.013)	0.084*** (0.025)	0.058*** (0.022)

Robust standard errors are in parentheses

*, ** and *** denote significance at the 10%, 5% and 1% level, respectively. Data is from the National Bureau of Statistics.

Columns (3)–(7) reveal the marginal effects of migrant workers receiving different types of public employment services on different levels of HS. The marginal effects in Columns (3) and (4) have no evidence to suggest that VI and VG affect HS, consistent with the conclusion of the benchmark regression. Column (5) manifests that the probability of “very satisfied” of migrant workers receiving VT distinctly increases by 4.8% (*p* < 0.01), whereas that of “relatively satisfied”, “average” and “relatively dissatisfied” significantly decreases by 1.4%, 3.1% and 0.3% (*p* < 0.01). Column (6) shows that the probability of “very satisfied” of migrant workers receiving JD evidently increases by 8.4%, whereas that of “relatively satisfied”, “average” and “relatively dissatisfied” decreases significantly by 2.5%, 5.5% and 0.4%, respectively. Column (7) suggests that the probability of “very satisfied” of migrant workers receiving ORS increases by 5.8%, and that of HS as “relatively satisfied”, “average” and “relatively dissatisfied” decreases significantly by 1.7%, 3.8% and 0.3%, respectively. In general, public employment services significantly improve the probability of HS as “very satisfied” and reduces that of “relatively satisfied”, “average” and “relatively dissatisfied”. These results suggest that the HS of a portion of migrant workers has changed from “relatively satisfied”, “average” and “relatively dissatisfied” to “very satisfied”.

### 3.3 Robustness checks

#### 3.3.1 Subsample regression results

Table 4 shows the results of subsample regression which can be used for the robustness checks of the benchmark regression. In terms of age groups, the HS of migrant workers receiving PES has been significantly improved in old and new generations; the improvement of the old generation is higher than that of the new generation. Compared with the new generation, the old generation aged over 35 has lower physical fitness, whose health status is more sensitive to public employment services. In terms of gender, PES has distinctly enhanced the HS of male and female groups; the improvement of the male group is higher than that of the female group. In terms of marriage, PES has apparently improved the HS of unmarried and married groups; the improvement of the unmarried group is higher than that of the married group. Overall, the results of the subsample analysis confirm the robustness of the core view that PES has a significant positive impact on the self-rated health of migrant workers.

**Table 4:** Subdivided sample analysis (by age, gender and marriage).

Variable	Dependent variable: HS					
	Old	New	Female	Male	Unmarried	Married
	(1)	(2)	(3)	(4)	(5)	(6)
PES	0.126**	0.099**	0.107*	0.123***	0.227***	0.089**
	(0.058)	(0.048)	(0.061)	(0.046)	(0.079)	(0.042)
Control variable	YES	YES	YES	YES	YES	YES
N	2024	2764	1779	3009	1014	3774

Robust standard errors are in parentheses

*, ** and *** denote significance at the 10%, 5% and 1% level, respectively. Data is from the National Bureau of Statistics.

#### 3.3.2 Substitution of the dependent variable

We adopt the method of substituting dependent variables for robustness test that replaces the dependent variable HS with HS1, and employ the Probit Model for the regression analysis and calculate the marginal effect of the estimated coefficient. The results are shown in Table 5. Columns (1)–(7) illustrate that the marginal effects of PES, TPES, VT, JD and ORS on the HS1 are significantly positive, which are 0.046, 0.014, 0.062, 0.099 and 0.092, respectively. The results reveal that public employment services improve the health satisfaction of migrant workers mainly through VT, JD and ORS, which are consistent with the core view of the benchmark regression.

**Table 5:** Robustness test: Substitution of the dependent variable.

Variable	Dependent variable: HS1						
	(1)	(2)	(3)	(4)	(5)	(6)	(7)
PES	0.046***						
	(0.015)						
TPES		0.014***					
		(0.013)					
VI			0.004				
			(0.017)				
VG				0.019			
				(0.017)			
VT					0.062***		
					(0.016)		
JD						0.099***	
						(0.033)	
ORS							0.092***
							(0.028)
Control variable	YES	YES	YES	YES	YES	YES	YES
N	4788	4788	4788	4788	4788	4788	4788

Robust standard errors are in parentheses

*, ** and *** denote significance at the 10%, 5% and 1% level, respectively. Data is from the National Bureau of Statistics.

#### 3.3.3 PSM analysis

To eliminate the potential sample selection bias, this study adopts PSM for robust analysis, and the balance test is reported in Table 6. Before the match, most of the covariables had significant differences at 1% or 5% level between the treatment groups and control groups, except for gender, household registration, residence permit, political identity, east and middle. After the match, all the differences of covariables did not pass the significance test at the 5% level, indicating that no systemic differences are observed between the treatment groups and control groups. Therefore, the results of PSM are reliable.

**Table 6:** Balance test of the main variables.

Variable	Unmatched			Matched		
	treatment group	control group	t (p-value)	treatment group	control group	t (p-value)
Gender	0.636	0.625	0.71(0.479)	0.636	0.647	-0.60(0.549)
Age	35.639	36.936	-3.87(0.000)	35.639	35.664	-0.07(0.947)
Marriage	0.765	0.797	-2.49(0.013)	0.765	0.771	-0.41(0.683)
Household registration	0.959	0.954	0.75(0.454)	0.959	0.964	-0.70(0.486)
Education	11.236	10.759	4.48(0.000)	11.236	11.025	1.69(0.091)
Residence permit	0.587	0.601	-0.91(0.362)	0.587	0.603	-0.86(0.390)
Political identity	0.027	0.029	-0.32(0.749)	0.027	0.023	0.74(0.461)
Trailing relatives	0.018	0.029	-2.12(0.034)	0.018	0.015	0.59(0.552)
Medical insurance	0.765	0.652	7.76(0.000)	0.765	0.765	-0.05(0.963)
East	0.344	0.333	0.74(0.459)	0.344	0.329	0.81(0.417)
Middle	0.515	0.487	1.75(0.080)	0.515	0.538	-1.19(0.234)
West	0.125	0.163	-3.28(0.001)	0.125	0.118	0.53(0.598)
Manufacture	0.451	0.372	5.06(0.000)	0.451	0.444	0.39(0.700)
Service	0.542	0.624	-5.3(0.000)	0.542	0.550	-0.42(0.672)

Table 7 shows the results of the PSM analysis. Firstly, using the nearest neighbour matching (*k* = 1), the HS of the treatment group receiving PES is significantly higher than that of the control group not receiving PES. The ATT is 0.064 (*p* < 0.05), denoting that PES improves the HS of migrant workers, which proves the robustness of the baseline regression results. We also adopt the methods of radius matching, kernel density matching and local linear regression matching to estimate the ATTs, which range from 0.06 to 0.069 (*p* < 0.05), confirming the robustness of the core view.

**Table 7:** Results of the PSM analysis (PES).

Matching method	Nearest neighbor matching	Radius matching	Kernel density matching	Local linear regression matching
Treatment group	4.177	4.177	4.177	4.177
Control group	4.113	4.117	4.109	4.108
ATT	0.064***(0.029)	0.068*** (0.021)	0.068*** (0.021)	0.069** (0.029)
Control variable	YES	YES	YES	YES

Standard errors are in parentheses

** and *** denote significance at the 5% and 1% level, respectively.

To further examine the robustness effects of different types of public employment services on the HS, we utilise the nearest neighbour matching (*k* = 1) to calculate the ATTs. The results are shown in Table 8. The ATTs of VI and VG are 0.042 and -0.004 respectively, but not passing the 5% significance level, which illustrates that VI and VG have no effect on the HS of migrant workers. The ATTs of VT, JD and ORS are respectively 0.105, 0.132 and 0.108. These values pass the significance level test of 5% and indicate that VT, JD and ORS significantly improve the HS of migrant workers. The above results demonstrate that the improvement of public employment services on HS is mainly derived from VT, JD and ORS, consistent with the conclusion of the baseline regression.

**Table 8:** Results of the PSM analysis (different types of public employment services).

Matching variable	Treatment group	Control group	ATT	Control variables
VI	4.139	4.097	0.042(0.034)	YES
VG	4.145	4.149	-0.004(0.034)	YES
VT	4.199	4.094	0.105***(0.031)	YES
JD	4.272	4.140	0.132***(0.058)	YES
ORS	4.229	4.121	0.108**(0.052)	YES

Standard errors are in parentheses

** and *** denote significance at the 5% and 1% level, respectively.

## 3.4 Mechanism analysis

In fact, migrant workers who have received public employment services may attach more attention to whether enterprises provide health care for them, such as medical insurance (MI), injury insurance (II) and other rights. Table 9 shows that the proportion of medical insurance and injury insurance purchased by enterprises for migrant workers is 69.2% and 68.5%. Among them, the proportion of medical insurance of participants is 77.7%, which is significantly higher than that of non-participants (65.9%), with a difference of 11.8%. The proportion of injury insurance of participants is 78.1%, which is significantly higher than that of non-participants (64.7%), with a difference of 13.4%. It indicates that public employment services may affect the health status of migrant workers by the mechanism of medical insurance and injury insurance.

**Table 9:** Differences in health insurance between participants and non-participants.

Insurance type	Total sample	Participants	Non-participants	Mean difference
Medical insurance	0.692	0.777	0.659	0.118*** (0.013)
Injury insurance	0.685	0.781	0.647	0.134*** (0.013)

Standard errors are in parentheses

** and *** denote significance at the 5% and 1% level, respectively.

We conduct mechanism test by using mediating variables of medical insurance and injury insurance, and the results are shown in Table 10. The column (1) confirms that the total effect equation of the impact of PES on the HS of migrant workers in the absent of mediating variables significantly exists. Column (2) examines the impact of PES on the mediator of MI, and the estimated coefficient is significantly positive, indicating that PES increases the proportion of medical insurance of migrant workers. Column (3) adds medical insurance (MI) into the total effect equation for estimation, and the estimated coefficient of MI is significantly positive. Hence, the results columns (1)-(3) proves that PES exerts a significantly positive effect on HS by improving the proportion of MI of migrant workers. Similarly, the results of columns (4)-(6) demonstrates that Injury insurance (II) is the pivotal mediating variables between the PES and HS, the positive effect of PES on the HS by improving the proportion of II of migrant workers.

**Table 10:** Mechanism test: Medical insurance and injury insurance.

variable	(1)	(2)	(3)	(4)	(5)	(6)
	HS	MI	HS	HS	II	HS
PES	0.122***	0.270***	0.116***	0.122***	0.315***	0.105***
	(0.036)	(0.047)	(0.037)	(0.036)	(0.045)	(0.037)
MI			0.095**			
			(0.042)			
II						0.191***
						(0.041)
Control variable	YES	YES	YES	YES	YES	YES
N	4788	4788	4788	4788	4788	4788

Standard errors are in parentheses

** and *** denote significance at the 5% and 1% level, respectively.

## 4. Discussion

The healthcare of migrant workers is the cornerstone of new-type urbanisation, high-quality development and social stability in the new era of China. Although existing studies have discussed the health issues of migrant workers, they have not addressed whether China’s public employment service system plays an important role in the health satisfaction of migrant workers. On the basis of the monitoring data of migrant workers in Shanghai from 2015 to 2020, this study, for the first time, therefore explores the impacts of public employment services on the self-rated health of migrant workers in China. The Ordered Probit Model, PSM and Mediating Effect Model are used to conduct empirical tests. The study illustrates an apparently positive correlation between public employment service and the HS of migrant workers; the more types of public employment services migrant workers have received, the higher their health satisfaction will be. The results are consistent with search-matching theory [33–35]. The free recruitment information provided by the public sector of employment services for migrant workers can reduce their search costs, contributing to the increase of the matching probability between migrant workers and safe and healthy jobs. Moreover, from the perspectives of the types of public employment services, VT, JD and ORS are the key to improve the health satisfaction of migrant workers. VT and JD can promote the human capital of migrant workers, such as skills, education or experience, which are conducive to enhancing their competitiveness in applying for high-quality jobs with better medical security. In addition, the mechanism analysis indicates that public employment services improve the self-rated health of migrant workers by the mediators of medical insurance and injury insurance.

Under the current impact of the COVID-19 pandemic, the health statuses of migrant workers are threatened. This study provides a new perspective from public employment services to safeguard the health statuses of migrant workers, which is enlightening for perfecting China’s public employment service system. First, the government should increase the fiscal support in the field of public employment services, strengthen the construction of institutional premises and professional staffing and solidify the foundation of the public employment service system. At present, China’s public employment service institutions are confronted with serious shortages of funds and professionals compared with developed countries. Second, the public sector must prioritise the assurance of public employment services for those with employment difficulties. With limited public resources at present, public departments should focus on the disadvantaged group of migrant workers and prioritise public employment services for this group. Therefore, strengthening the VT of grassroots practitioners in public employment services and improving the efficiency and quality of these services are required. Third, the public sector should strengthen the application of information technology in public employment services. Utilising the interactive and instantaneous advantages of big data and Internet technology promotes the extension and expansion of public employment services, realises the online processing of services and improves the efficiency of employment information collection and dissemination. Meanwhile, the public sector must establish the database and improve the information management, analysis and application system for public employment services.

## 5. Limitations and future research

This study still has several limitations. Firstly, we use the mixed cross-sectional data, rather than the tracking data. The estimated results may reflect correlation, rather than causation. Future studies may use tracking data to accurately identify the causal effect between public employment services and health satisfaction. Secondly, the core explanatory variable in this study adopts a dummy variable due to data limitations. This variable assumes that the public employment services received by migrant workers are identical, which may lead to inaccurate estimation results. Future research should collect data on the frequency and duration of migrant workers receiving public employment services. Thirdly, the research area is Shanghai, which has limited sample representation. Our policy implications can be used for reference in the Yangtze River Delta region or typical large cities that input migrant workers. However, extending the implications to the whole country requires caution. Finally, the COVID-19 pandemic may be one of the factors affecting the self-rated health of migrant workers. However, its interference cannot be effectively controlled due to data limitations.

## 6. Conclusion

This study reveals the significant positive correlation between public employment services and the self-rated health of migrant workers. The results support the search-matching theory, indicating that the public sector of employment services can reduce search costs for migrant workers, thereby increasing the matching probability with safe and healthy jobs. In order to check the robustness and verify the reliability of the results, we adopt the methods of subdividing the samples, substituting the dependent variable and PSM. We find that the results are robust and reliable.

## Appendix

**Table 11:** Definition of variables.

Variable	Definition
Dependent variable	
HS	Health satisfaction. Five categories: from very dissatisfied = 1 to very satisfied = 5.
**Core explanatory variable**	
PES	Have you received public employment services? Yes = 1, no = 0
TPES	How many types of public employment services have you received?
VI	Have you received vocational introductions? Yes = 1, no = 0
VG	Have you received vocational guidance? Yes = 1, no = 0
VT	Have you received vocational training? Yes = 1, no = 0
JD	Have you received job development? Yes = 1, no = 0
ORS	Have you received any other employment services? Yes = 1, no = 0
**Control variable**	
Gender	Male = 1,female = 0
Age	Respondents’ age
Marriage	Married = 1,unmarried = 0
Household registration	Urban = 1, rural = 0
Education	Education years of the respondent
Residence permit	Do you have a residence permit? Yes = 1,no = 0
Political identity	Party member = 1, else = 0
Trailing relatives	Are you the trailing relative? Yes = 1,no = 0
Medical insurance	Does the company pay for your medical insurance? Yes = 1,no = 0
East	Domicile in the east = 1, else = 0
Middle	Domicile in the middle = 1, else = 0
West	Domicile in the west = 1, else = 0
Manufacture	Engaged in manufacturing industry = 1, else = 0
Service	Engage in service industry = 1, else = 0

Data is from the National Bureau of Statistics.

## Supporting information

S1 Data (XLS)
